# Heat Transfer Analysis of 3D Printed Wax Injection Mold Used in Investment Casting

**DOI:** 10.3390/ma15196545

**Published:** 2022-09-21

**Authors:** Bartłomiej Burlaga, Arkadiusz Kroma, Przemysław Poszwa, Robert Kłosowiak, Paweł Popielarski, Tomasz Stręk

**Affiliations:** 1 Division of Virtual Engineering, Institute of Applied Mechanics, Faculty of Mechanical Engineering, Poznan University of Technology, 60-965 Poznan, Poland; 2 Division of Foundry and Plastic Working, Institute of Materials Technology, Faculty of Mechanical Engineering, Poznan University of Technology, 60-965 Poznan, Poland; 3 Division of Aeronautical Engineering, Institute of Thermal Energy, Faculty of Environmental Engineering and Energy, Poznan University of Technology, 60-965 Poznan, Poland; 4 Division of Technical Mechanics, Institute of Applied Mechanics, Faculty of Mechanical Engineering, Poznan University of Technology, 60-965 Poznan, Poland

**Keywords:** casting, wax injection molds, resin, cooling, finite element method (FEM), heat transfer

## Abstract

Investment casting is one of the precise casting methods where disposable wax patterns made in wax injection molds are used to make a casting mold. The production capacity of precision foundry is determined by the time taken for producing wax patterns, which depends on the time taken for wax solidification. Wax injection molds are usually made of aluminum or copper alloys with the use of expensive and time-consuming computer numerical control (CNC) processing, which makes low-volume production unprofitable. To reduce these costs, the authors present a heat transfer analysis of a 3D printed wax injection mold. Due to the low thermal conductivity of the photopolymer resin, the influence of different cooling channels’ shapes was investigated to improve the time of the manufacturing process. Transient thermal analysis was performed using COMSOL software based on the finite element method (FEM) and included a simulation of wax injection mold cooling with cold air (−23 °C), water, and without cooling. The analysis showed that use of cooling channels in the case of photopolymer material significantly reduces the solidification time of the sample (about 10 s shorter), and that under certain conditions, it is possible to obtain better cooling than obtained with the aluminum reference wax injection mold (after approximately 25–30 s). This approach allows to reduce the production costs of low-volume castings.

## 1. Introduction

The competitive production of products is very often directly related to the ability to quickly and efficiently respond to market changes. These changes may result not only from the increased requirements and expectations of customers for products, but also from geopolitical situation, such as the related prices of raw materials necessary for the production of products. Redesigning products to fully meet specific requirements in many cases is not an easy, quick, and cheap process. As a result, enterprises unprepared for such activities lose their position on the market [1,2]. To reduce the cost and time-consuming response, tools such as rapid prototyping, computer aided design (CAD), and computer aided manufacturing (CAM) systems [3], or simulations based on advanced methods of numerical solving of boundary problems, such as the Finite Element Method (FEM), can be used [4,5,6,7].

Rapid prototyping and tooling is a set of technologies (usually additive) that allow, thanks to the use of CAD models, to quickly produce physical products and tooling [8]. The use of additive techniques enables the production of objects with complex and irregular shapes, which is very often impossible in the case of other technologies. However, due to the high level of integration of computer systems into production processes in the era of Industry 4.0 [9], the term rapid prototyping and tooling should be considered as a broader concept, because its components are often directly or indirectly related to other forms of production, such as computer numerical control (CNC) machining (through the integration of CAD/CAM environments) [10], reverse engineering (3D scanning) [11] or investment casting (making disposable casting patterns) [12,13].

Investment casting is a method that allows to make complex shaped castings thanks to the use of disposable casting patterns [14]. In the case of mass production, these models are made of wax mixtures [15], which are formed by injecting a liquid or semiliquid wax mixture into a reusable wax injection mold. The wax injection molds due to the high rate of heat conduction are mainly made from copper and aluminum alloys [13] with the use of CNC machining, but in prototypical and unit production, thanks to the use of patterns (master model), wax injection molds can be made of rubber or silicones [16,17].

The application of 3D printing technology in the injection molding processes can be seen in the plastics industry. For many years, the plastic processing industry has been using exchangeable inserts of injection molds to improve versatility and reduce the cost of tooling [18]. Manufacturers of 3D printing materials and printers are increasingly trying to develop such materials that can be used in the injection processes as much as possible. The leader in this field is Stratasys Ltd., which uses its patented PolyJet photopolymer 3D-printing technology to manufacture 3D printed inserts for injection molds. However, due to the high temperature of injection of plastics (200–300 °C), printed 3D injection inserts are subject to degradation and, depending on the geometry and process, provide from several to about 100 injection moldings [19,20].

In addition, in the case of prototype or unit casting production, it is possible to avoid using the wax injection molds, but this entails the need to introduce additional materials with different parameters to the production system, which, for example, increase the annealing time of the casting mold. The single pattern (casting model) can be 3D printed from materials characterized by low ash-free burnout [13,21], e.g., high impact polystyrene (HIPS), acrylonitrile butadiene styrene (ABS), photopolymer casting resin (ash content thermogravimetric analysis (TGA) 0.0–0.1%) [22]. The most commonly used 3D printing techniques for disposable casting patterns are: fused deposition modelling (FDM), stereolithography (SLA) [23], SLS (selective laser sintering) [24], daylight polymer printing (DPP) [25] and digital light processing (DLP) [26].

**Figure 1:**
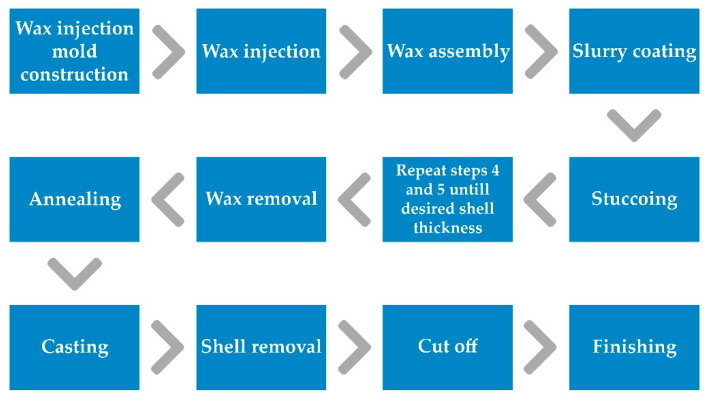
The block diagram of the investment casting process.

After making the casting patterns, in the next stage of investment casting process the patterns (wax or plastic) are combined into sets, which are then used to make the casting mold. The casting mold, depending on the production volume and the alloy temperature, is made of ceramic mixtures based on hydrolyzed ethyl silicate or gypsum [27]. To remove the patterns from the cavity of the mold and to allow it to be filled with a liquid alloy, the prepared mold is placed in the furnace, where it is subjected to the annealing process. As a result of this process, the patterns melt and flow out (wax patterns) or burn out (plastic patterns), creating an empty cavity into which can be poured with a liquid alloy. The block diagram of the investment casting process is shown in Figure 1.

To avoid costly and time-consuming verification processes of the introduced changes, it is possible to use various simulation environments depending on the described problem. This study focused on the heat transfer and fluid flow phenomena, which can be approximated with partial differential equations. The high complexity equations require efficient solving methods. The finite element method (FEM) was chosen to obtain a solution in the presented computational model. FEM is used very often as a tool which can help to show important elements of complicated processes and engineering problems in heat transfer from the estimation of thermo-mechanical parameters of material to topology optimization of the thermal properties of materials [28,29,30].

A typical workflow of this method can be divided into two stages: dividing the problem into subdomains (each one is represented by element equations) and recombination sets of subdomain’s equations for a final solution. The initial values and boundary conditions are necessary to obtain numerical solutions. The COMSOL Multiphysics software (COMSOL Inc., Stockholm, Sweden) has been used with modules for solving governing equations for heat transfer in fluids and solid bodies with naturally occurring phenomena (including conduction, radiation, and convection).

This study shows the cooling process of a 3D printed photopolymer wax injection mold with different types of cooling channels. The application of the photopolymer wax injection mold required experimental tests in order to obtain thermal parameters of the used materials. Moreover, to achieve the best possible results of simulation, it was decided to perform a heat transfer analysis for two different cooling media, the water and cold air (−23 °C), with flow rate of: 0.0003 kg/s, of 0.0006 kg/s, and 0.001 kg/s. The findings suggest the applicability of this technology in the foundry industry to make short series of castings.

## 2. Materials and Methods

### 2.1. Theoretical Background

#### 2.1.1. Fourier Law

Heat transfer phenomena are described by rate equations, which are used to calculate the amount of energy being transferred per unit time. For the steady-state heat conduction problem (when the system is in equilibrium), the rate equation is known as Fourier’s law and is described as Equation (1) [31]:

(1) q=−k∇T

where heat flux *q* (W/m^2^) is the heat transfer rate in the per area unit perpendicular to the direction of transfer, and it is proportional to the material’s thermal conductivity *k* (W/m·K), temperature gradient ∇T (K/m). The *T*(**x**) [K] is the scalar temperature field) [26]. This equation was formulated by Joseph Fourier [32] in 1822, who concluded that the heat flux resulting from thermal conduction is proportional to the magnitude of the temperature gradient and opposite to it in sign. This equation is widely used for quasi-equilibrium processes, where the temperature distribution in the system is important. It is widely used in process engineering (reactors, heat exchangers) [33] and environmental engineering (heating, ventilation, air conditioning) [34].

#### 2.1.2. The Navier–Stokes Equation

The motion of viscous fluid substances can be described by certain partial differential equations called Navier-Stokes Equations, which were developed over several decades by French engineer and physicist Claude-Louis Navier and Anglo-Irish physicist and mathematician George Gabriel Stokes in the XIX century [35].

The Navier–Stokes equations mathematically describe the conservation of momentum and mass for Newtonian fluids. Sometimes, they are accompanied by an equation of state relating to pressure, temperature, and density. They were developed by applying Isaac Newton’s second law to fluid motion, along with the assumption that the stress in the fluid is equal to the sum of a diffusing viscous term (proportional to the gradient of velocity) and a pressure term—hence describing the viscous flow [35].

The Navier–Stokes equations are used by scientists and engineers to describe the physics of many phenomena. They are widely used for engineering problems (water flow in pipes, heat exchangers, molds, airflow around bodies, such as airplanes and cars, HVAC, turbomachinery design) and scientific modeling (ocean currents, weather models, air mass movement, air pollution distribution). For the motion of an incompressible, constant density, viscous fluid, the Navier–Stokes equations have the form of Equation (2) [35]:

(2) $$\begin{array}{c} \frac{\partial v}{\partial t}+\left(v\cdot \nabla \right)v=-\frac{1}{\rho }\nabla p+\nu \nabla^{2}v,\\ div\left(v\right)=0, \end{array}$$

where ***v***(**x**,t) is the velocity vector, *p*(**x**,t) is pressure, and the constants *ρ* (kg/m^3^) and *ν* (m^2^/s) are the density and kinematic viscosity, respectively [28].

#### 2.1.3. Nonisothermal Flow

Many industrial processes are non-isothermal processes, where the temperature changes during the process and has a significant influence on the process flow (such as plastics injection molding and extrusion, heat treatment of steel, and food processing) [36,37,38,39]. For industrial processes, one of the crucial aspects is the length of the cooling phase, as it influences the time needed to finish the manufacturing process [40]. The transient thermal problems cannot be described with Fourier law, as it is used for steady-state phenomena. The transient temperature response is related to the overall energy balance of the investigated system. This balance is determined by the rate of heat loss at the surface ${\dot{E}}_{out}$ (J/s) to the rate of change of the internal energy ${\dot{E}}_{st}$ (J/s) (Equation (3)) [31]:

(3) $$-{\dot{E}}_{out}={\dot{E}}_{st}\;$$

The rate of heat loss can be described by Newton’s law of cooling and the rate of change of internal energy is described by temperature change (Equation (4)) [31]:

(4) $$-h\;A_{s}\left(T-T_{\infty }\right)=\rho Vc\frac{dT}{dt}\;\;$$

where, *h* (W/(m^2^ K)) denotes the convection heat transfer coefficient, *A_s_* (m^2^) the area normal to the heat transfer, $T_{s}$ (K) the surface temperature, $T_{\infty }$ (K) the ambient or fluid (in case of cooling channels) temperature, ρ (kg/m^3^) density, *V* (m^3^) volume, and *c* (J/kg K) specific heat [31].

By substituting $T-T_{\infty }$ with temperature difference θ and integrating the equation from the initial condition, Equation (5) [31] can be obtained:

(5) $$\frac{\theta }{\theta_{i}}=\frac{T-T_{\infty }}{T_{i}-T_{\infty }}=\exp\left(-\frac{hA_{s}}{\rho Vc}t\right)\;$$

The inverse value of the exponential term is often called the thermal time constant τ (Equation (6)) [31]:

(6) $$\tau =\frac{\rho Vc}{hA_{s}}=\frac{1}{hA_{s}}\rho Vc=R_{t}C_{t}\;$$

where *R_t_* and *C_t_* are the resistance to convection heat transfer and the lumped thermal capacitance of the material, respectively. Any increase in *R_t_* or *C_t_* will cause a solid to respond more slowly to changes in its thermal environment. Equation (5) and its derivatives are widely used for estimating cooling time in industrial processes.

Ignoring viscous dissipation and pressure work, the heat equation for fluid can be written in the form of Equation (7) [31]:

(7) $$\rho_{f}c_{f}\left(\frac{\partial T}{\partial t}+\left(v\cdot \nabla \right)T\right)+\nabla \cdot \left(-k\nabla T\right)=0\;$$

If the velocity is set to zero, the equation governing purely conductive heat transfer in solid is obtained from Equation (8) [31]:

(8) $$\rho_{s}c_{s}\frac{\partial T}{\partial t}+\nabla \cdot \left(-k\nabla T\right)=0\;$$

where $\rho_{f},\;\rho_{s}$ are densities for fluid and solid, respectively, and $c_{f},\;c_{s}$ are specific heat at constant pressure for fluid and solid, respectively.

### 2.2. Experimental Values of Material Properties

Obtaining the key material data is one of the most important, necessary steps before starting the simulation. In the case of the heat transfer analysis, these are: thermal conductivity and specific heat, the values of which can be determined or taken from the tables of the simulation software. In the analyzed case, it was necessary to experimentally determine the necessary parameters due to the lack of information from the manufacturer.

#### 2.2.1. Determination of Thermal Conductivity

**Figure 2:**
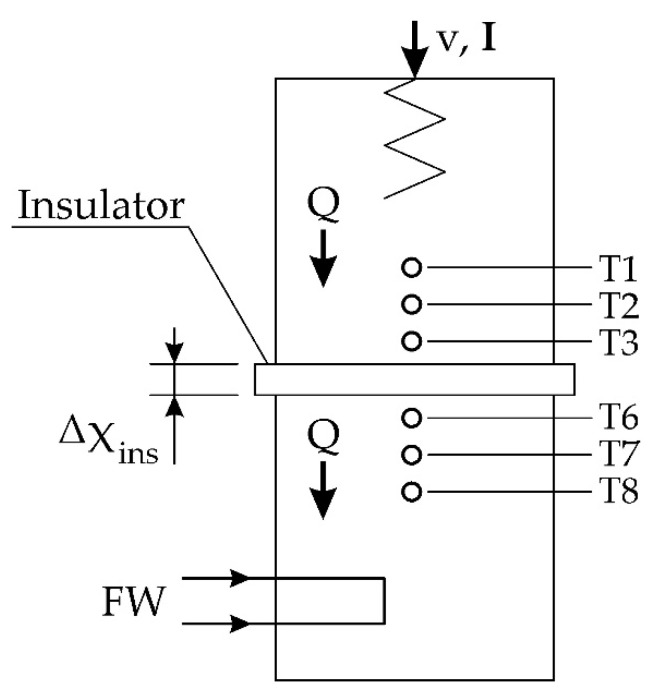
The application of poor conductors. Where: T (1,2,3,6,7,8) thermocouple, $\Delta x_{red}\;\mathrm{or}\;\Delta x_{ins}$ is a thickness of measure sample, U is voltage, A—ampere, Q is heat flux, FW—flow of cooling water.

The temperature difference was measured using a known steady flow of heat across a specimen of insulating material [41] and the Fourier rate (Equation (1)) to calculate the thermal conductivity of the material, as shown at Figure 2.

**Figure 3:**
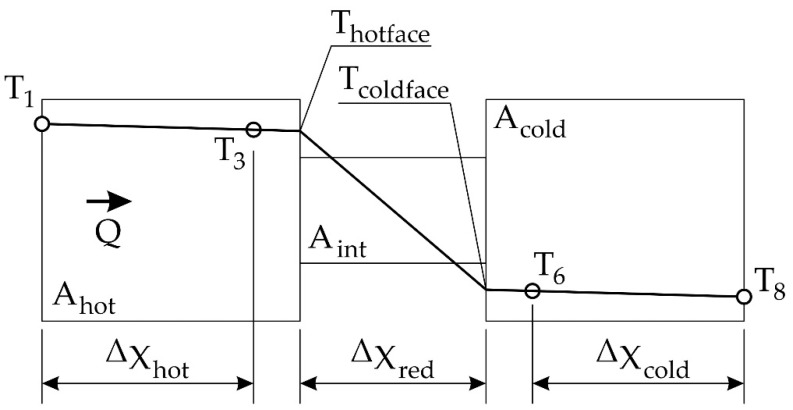
Theoretical temperature distribution in cross-sections. Where: X—length of the sections [m], A—is a cross-section [m^2^], T (1,3,6,8)—temperature at measure points, $T_{hotface},T_{coldface}$—calculated temperature at boundary of the layer.

The heating section represented by thermocouple T1, T2, and T3 is manufactured from 25 mm diameter cylindrical brass bar with a cartridge type electric heating element installed at one end. The cooling section represented by thermocouple T6, T7, and T8 is manufactured from 25 mm diameter cylindrical brass bar to match the heating section and cooled at one end by water (FW) passing through galleries in the section. The heated and cooled sections are clamped tightly together with the poor conductors in between to create a composite bar with the insulated disk of unknown thermal conductivity sandwiched between two brass sections. The theoretical temperature distribution in cross-sections is shown in Figure 3.

Insulators have very low values of thermal conductivity which means that only a small amount of heat will pass through the material even though a high temperature difference may exist across its two faces. Because of the low value of k for an insulator, the dimension x must be small [42]. The heat flow is calculated from the temperature gradient for the hot (9) and cold (10) parts. This is possible thanks to the known thermal conductivity of the heater and cooler, as well as the position of the thermocouples and the temperature value.

Heat flow of heater calculated from Equation (9) [42]:

(9) $$Q_{h}=k_{heater}\frac{T_{1}-T_{3}}{\Delta x_{1-3}}\;$$

Heat flow of cooler calculated from Equation (10) [42]:

(10) $$Q_{c}=k_{cooler}\frac{T_{6}-T_{8}}{\Delta x_{6-8}}\;$$

where *k* is thermal conductivity of the heater and cooler.

It follows from the energy balance; Equation (11) [42]:

(11) $$Q=Q_{c}=Q_{h}\;$$

After calculating the heat flux, the temperature at the boundary of the layers is determined according to Equation (1). The values of these temperatures ($T_{hotface},T_{coldface})$ are necessary to determine the thermal conductivity of the tested material according to Equations (12) and (13) [42].

Heat flow is calculated:

(12) $$Q=k_{i}\frac{T_{hotface}-T_{coldface}}{\Delta x_{red}}\;$$

Therefore:

(13) $$k_{i}=\frac{Q}{A_{int}\left(T_{hotface}-T_{coldface}\right)}\;$$

#### 2.2.2. Determination of Specific Heat

**Figure 4:**
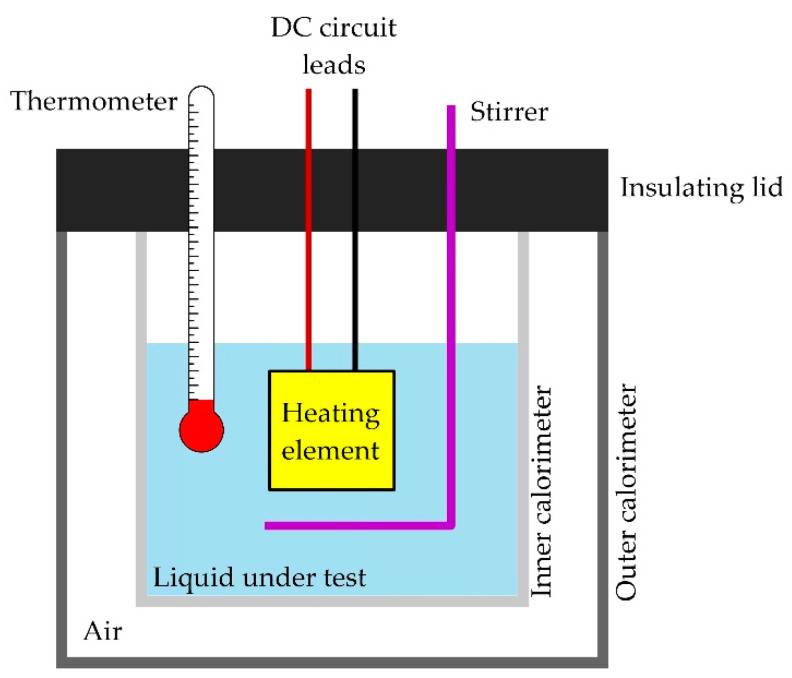
Schematic diagram of the calorimeter.

To determine the heat capacity, the energy balance method [43] was used. In this respect, two measurement techniques can be distinguished, as shown in the figure. Both measurements are made using a calorimeter or a bicalorimeter. The construction of the calorimeter is shown in Figure 4.

The first measurement techniques is based on the Joule–Lenz (Equation (14)) [44,45] heat to the heat energy factor, which is emitted from the conductor of electrical energy (Equations (15) and (16)) [44,45], and voltages from Ohm’s law (Equation (17)) [44,45].

(14) $$Q=≅I^{2}Rt\;$$

(15) P=U·I 

(16) $$P=\frac{U\;}{R}\;$$

(17) $$P=I^{2}R\;$$

Including the thermal energy of the measured liquid (Equation (18)) [44] is:

(18) Q=mcΔT 

After converting to specific heat *c* (Equation (19)) [44] and taking into account the Joule–Lenz heat:

(19) $$c=\frac{I^{2}R\;t}{m\;\Delta T}\;$$

The second method [46] is used to balance the energy (Equation (20)) [44,46] of two miscible liquids in a calorimeter vessel.

(20) $$Q_{c}=Q_{k}\;$$

The solid body of known mass $m_{c}$ is heated to the boiling point of water (*T_c_*), while the calorimeter has prepared water of mass m_w_ and temperature *T_p_*. The bold form of the notation indicates that the value is represented as a vector. The measurement is carried out by taking the heated body out of the steam bath and then placing it in a calorimeter filled with water. The last step is the stabilization of the end temperature *T_k_*. The heated body gives off heat (Equation (21)) [44], which is completely absorbed by the calorimeter and water (Equation (22)) [44].

The given heat is therefore:

(21) $$Q_{c}=m_{c}\;c\;\left(T_{c}-T_{k}\right)\;$$

where *c* denotes wanted heat capacity

(22) $$Q_{k}=\left(m_{w}\;c_{w}+m_{k}\;c_{k}\right)\;\left(T_{k}-T_{p}\right)\;$$

Taking into account the heat of the calorimeter and the tested body in the energy balance, an unknown heat capacity can be determined according to the Equation (23) [44].

(23) $$c=\frac{\left(m_{w}\;c_{w}+m_{k}\;c_{k}\right)\;\left(T_{k}-T_{p}\right)}{m_{c}\;\left(T_{c}-T_{k}\right)\;}.\;$$

where *c* denotes wanted heat capacity, $c_{k}$ heat capacity of calorimeter, $c_{w}$ heat capacity of water, $m_{c}$ mass of investigated body, $m_{k}$ mass of calorimeter, $m_{w}$ mass of water, *T_c_* temperature of investigated body, *T_p_* temperature in calorimeter at the beginning, and *T_k_* temperature in calorimeter at the end.

### 2.3. Computational Model

#### 2.3.1. The Finite Element Method (FEM)

The finite element method (FEM) allows engineers and scientists to obtain approximate solutions to differential equations. In such an analysis, multiple variables must satisfy these equations in any part of the computational domain and satisfy specific conditions on boundaries. To proceed with the developing solution, the values of mentioned variables need to be defined in specific areas of the domain, most often on its boundaries (boundary conditions; BC). For example, in heat transfer analysis, the heat flux or temperature can be defined, and likewise, the fluid velocity is one type of BC used in fluid dynamics simulations [4,47,48].

Often, boundary value problems are called field problems. The dependent variables of interest are governed by differential equations and are called field variables. If all field variables can be determined at every point in the analyzed domain and the governing equation is satisfied exactly at each point, the exact mathematical solution can be obtained. The analytical approach is possible for simple domains. When the complexity increases, other methods must be applied. The most widely used is FEM, a numerical technique able to obtain approximate results with good accuracy [4,47].

The general concept of FEM is based on dividing the domain into small elements with finite volume. Depending on the number of dimensions in the analyzed problem, one-, two- and three-dimensional elements can be generated. The vertices of the element are called nodes and are connected by edges. The internal nodes are not connected to other elements contrary to external nodes placed on boundaries. The values of field variables are approximately calculated in nodes. The values between nodes are computed by interpolation of nodal values with help of known shape functions. If certain mathematical requirements are satisfied, a finite element solution converges to the exact solution of the problem [47,48].

#### 2.3.2. Model and Wax Injection Mold

**Figure 5:**
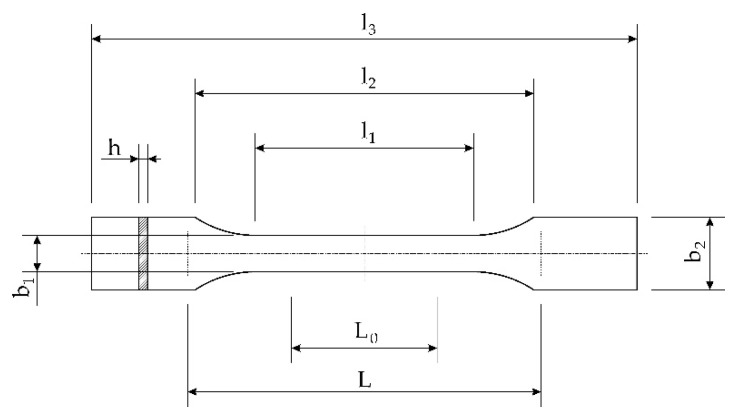
The geometry of the sample ISO EN ISO 527-2:2012 [49] (Table 1).

The SLA 3D printed injection mold made of Rigid 10K resin (Formlabs Ltd., Somerville, MA, USA) was analysed. The geometry (Figure 5) has been designed to allow the production of 1BA tensile strength test sample by the ISO EN ISO 527-2:2012 standard (Table 1) [49]. The Rigid 10K resin was selected for analysis because it is characterized by the highest thermal conductivity of all the photopolymer resins offered by Formlabs. For the test, a wax mixture of 118174 Freeman Flakes Wax—Super Pink (Freeman Manufacturing & Supply Company, Avon, OH, USA) characterized by the quickest solidification time in the Freeman Flake Wax line was used. Quick solidification is an important factor due to the low thermal conductivity of polymer injection mold compared to the aluminium.

**Figure 6:**
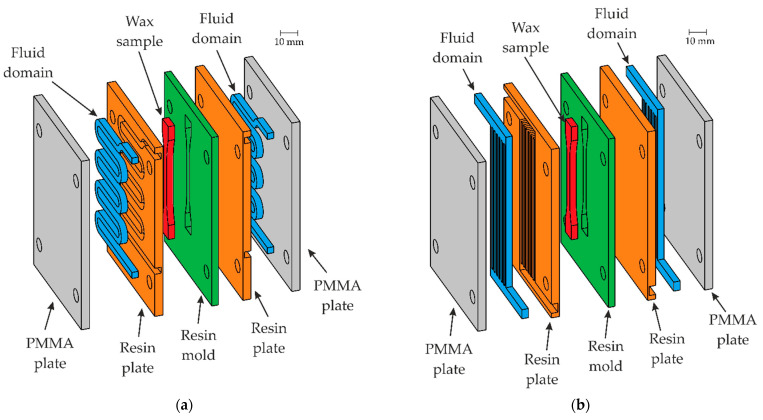
Exploded view of wax injection mold assembly: (**a**) with squared cooling channels (blue), (**b**) and finned cooling channels (blue).

**Figure 7:**
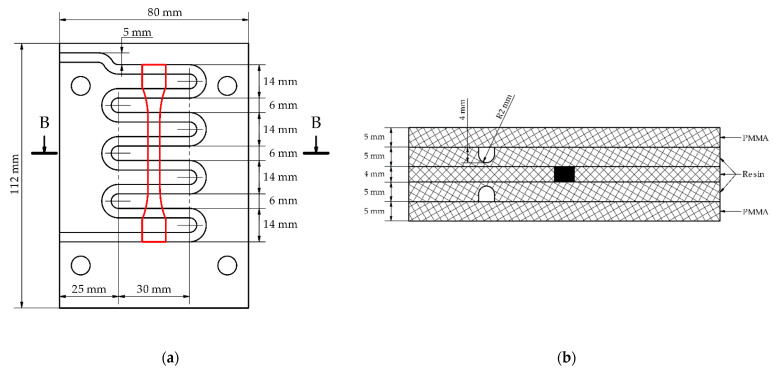

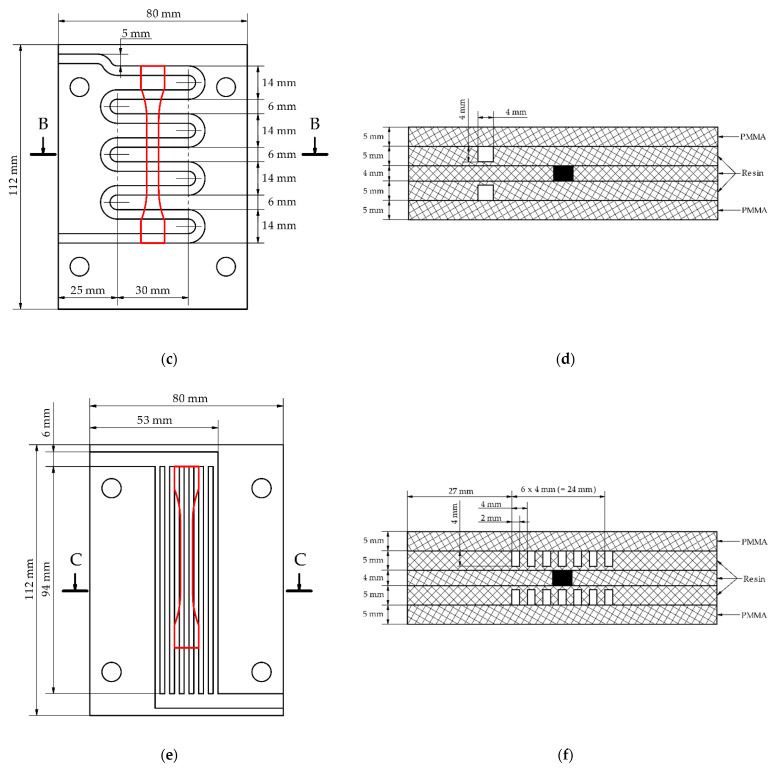
Variants of the wax injection mold geometry: (**a**) wax injection mold with rounded cooling channels, (**b**) cross-section of the rounded cooling channel, (**c**) wax injection mold with square cooling channels, (**d**) cross-section of the square cooling channel, (**e**) wax injection mold with finned cooling channels, (**f**) cross-section of the finned cooling channel. All dimensions in millimeters.

The wax injection mold layout consists of 5 plates. Figure 6 shows the exploded view of a wax injection mold with squared (a) and finned (b) cooling channels (blue). The 3 inner parts are responsible for the forming of the sample (the green one with a shape of the wax pattern, the two orange elements with cooling channels), with two polymethyl methacrylate (PMMA) insulators (grey). Detailed variants have been defined respectively (rounded, square, and finned cooling channels) in Figure 7.

**Figure 8:**
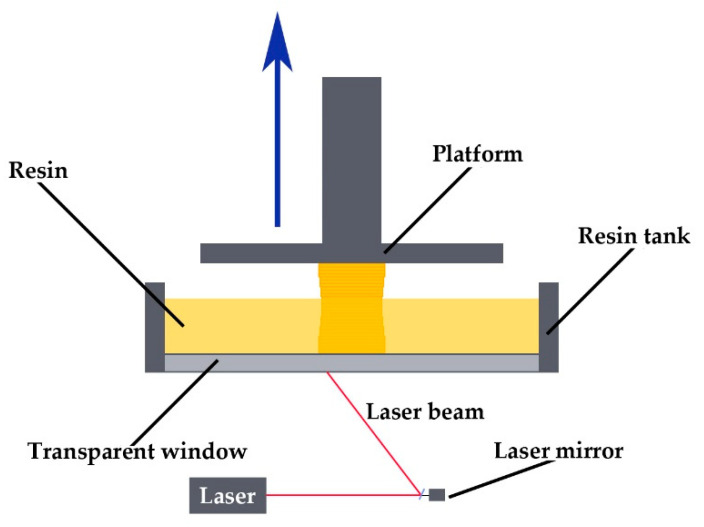
The diagram of the SLA process.

SLA is one of the most accurate 3D printing technologies. It uses photopolymer resins and allows to 3D print models with a single layer thickness of 25 μm. It consists in irradiating the liquid resin with a laser beam. As a result, the resin cure and forms a single layer in the cross-section of the final product. The next step is to lift the platform on which the printed model is created and repeat the process. Elements made using this technology, depending on the type of resin used, are characterized by a very good surface quality, high dimensional accuracy, and strength. The diagram of the technology is shown in Figure 8.

#### 2.3.3. Boundary Conditions

The boundary conditions in this study must be divided into two categories. The BCs of another one define the fluid flow.

##### Heat Transfer Boundary Conditions

To find a solution to differential equations governing these phenomena, the global ambient temperature must be defined. This is done by specifying an initial temperature of 293.15 K to all domains, but sample one. To obtain a higher level of convergence, the initial temperature of the sample is linearly ramped from room temperature to 338.15 K in 1 s. The initial values of cooling simulation are obtained from described temperature ramping stage and the wax domain is nonisothermal.

The thermal contact between plates is taken into consideration as boundary conditions defined between adjacent domains. The first one is applied between plastic plates as an equivalent thin resistive layer. According to the literature [50], it should be assumed that a thin, 20-um air gap exists between the mold elements in order to anticipate delamination of wax in cavity due to shrinkage and thermal contact. The thermal conductivity of this layer is calculated by computing software based on the temperature in each one of the time steps. The wax shrinks during the curing process. Due to this phenomenon, the value of thermal conductivity between model and wax injection mold changes with temperature and time. In the commercial software for injection molding simulations of polymeric materials, the constant value of heat transfer coefficient of thermal contact is used. The value depends on the phase of injection molding process and for cooling phase the value of 1250 (W/m^2^K) is used [51]. As the wax is chemically similar to polymeric materials (with potentially higher shrinkage), in this work, the mentioned value was used for the approximation of thermal contact.

**Figure 9:**
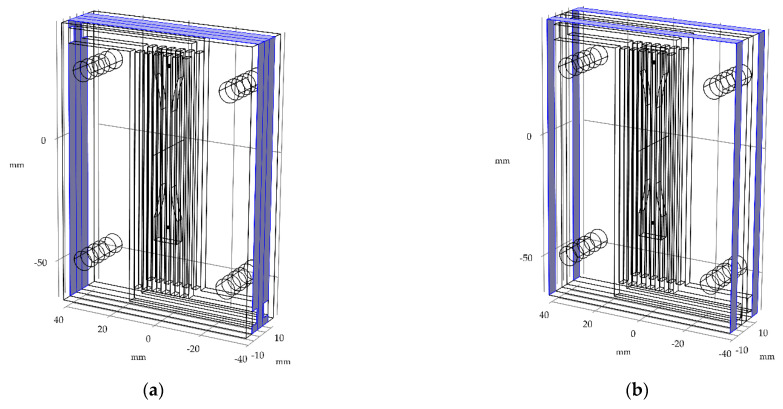
The surface emissivity: (**a**) the wax injection mold heat radiation surfaces, (**b**) the PMMA heat radiation surfaces.

The outer boundaries (Figure 9) have two different boundary conditions applied to them. The heat flux BC is used to simulate convective heat exchange with the surrounding air. The typical values of heat transfer coefficient in still air are between 3 and 15 W/(m^2^K). The higher values are observed in moving air, up to ca. 500 W/(m²K) depending on the temperature difference. The authors used 5 W/(m²K) to simulate nominal working conditions, i.e., still air with normal temperature. The other BC on the external boundaries is heat radiation, the rate of which depends, in great measure, on surface properties. The SLA resin parts (Figure 9a) have surface emissivity property set to 0.7 and the value for outer parts is 0.16 (Figure 9b).

The other external boundaries are assumed to be perfectly thermally insulated. This presumption is based on the physical model; these walls are isolated and a neglectable amount of heat flux is present.

All variants of the model (square-shaped channel, rounded channel, radiator-like channel, and without channels) have the same BC applied and defined the same way. The difference is in the addition (or lack) of cooling channels and cooling fluid.

##### Fluid Flow Boundary Conditions

The boundary conditions described in this paragraph are crucial for performing nonisothermal fluid flow simulation. This BC is applied exclusively to fluid domains.

**Figure 10:**
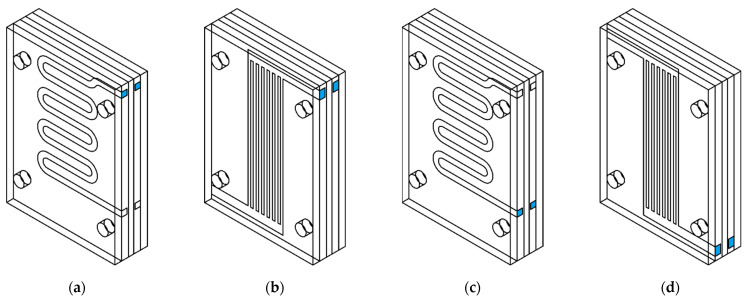
The cooling media inlets and outlets (respectively) for: (**a**,**c**) square and rounded channel, (**b**,**d**) finned channel.

Firstly, the inlet BC is defined. The boundary at which fluid enters domain is shown in Figure 10a for square and rounded channels and Figure 10b for the finned channel. In this simulation, the mass flow conditions on inlet boundaries depended on the type of fluid used for cooling. Three values were selected for air, which are 0.003 kg/s, 0.006 kg/s, and 0.01 kg/s. The highest value of fluid velocity with the lowest value of flow rate in square channels is ca. 80 m/s. This allows obtaining a turbulent flow, which is required to efficiently exchange heat [52], and 20 °C for water. For the second fluid, water, a different value was used. Due to the much higher viscosity, the inlet flow rate was decreased to 0.02 kg/s. In the case of liquid in cooling channels, Reynold’s number also was lower, and the character of flow changed to laminar. Additionally, on the same boundary, the temperature of the inflowing fluid, the air temperature, according to the capabilities of the cooling nozzle [53] was set to −23 °C.

On the other end of the cooling channel, a simple outlet BC was set, as shown in Figure 10c,d. This boundary condition allows setting external force or pressure that restricts flow. In the presented simulation, an open boundary without any flow resistance was applied.

The wall conditions of no-slip are applied to all other external boundaries. For simulations of channels and other enclosed flows, the velocity of fluid particles decreases with distance from the channel centerline and reaches zero at a specified boundary.

In total, three variants were simulated. In the first, the air enters the fluid domain with temperature of 250.15 K (or −23 °C) with three different non-zero flow rates as mentioned earlier. In the second variant of simulation, the air was replaced with water at 20 °C and the flow rate of 0.02 kg/s. The last variant is simulated without any flow and cooling channels, but with different materials to obtain reference values.

#### 2.3.4. Meshing

The mesh generation is important step in the process of setting up a simulation. In this study, the meshing tools used are part of Comsol Multiphysics version 5.3 software. The initial mesh was generated using “Fine” settings, then it was modified. The shape of finite element was set to tetrahedral for both solid and fluid domains. For the finned version, the maximum element size for laminar flow was equal to 3.00 mm and minimum size was set to 0.6 mm with defined maximum element growth rate of 1.15. The boundaries on the interface between fluid and solid, were meshed separately with following settings: maximum element size set to 1.0 mm, minimum element size set to 0.08 mm, and the maximum growth rate set to 1.1. Additionally, on mentioned boundaries, the two extra mesh layers were defined. The rest of the geometry, corresponding to solids, was meshed with different settings. The maximum element size was increased to 4.0 mm, the minimal element size was set to 0.4 mm and the maximum growth rate was also increased to value of 1.35. To evaluate correct mesh generation, the two quality measures were analyzed, i.e., the skewness of the elements and the volume of the element versus length. Both values were above the required minimum: the minimum skewness was 0.41 and lowest value of second measure was equal to 0.43.

**Figure 11:**
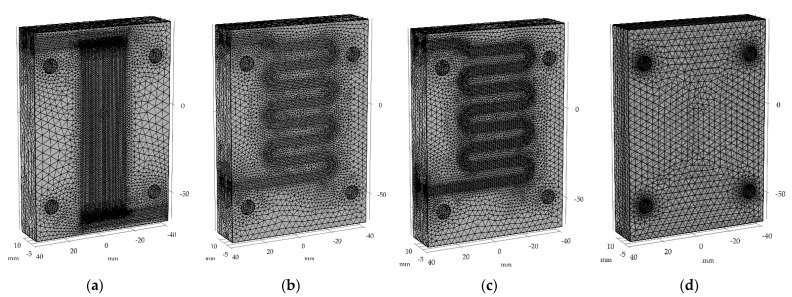
The final meshes for wax injection molds with: (**a**) finned channels, (**b**) square channel, (**c**) round channel, (**d**) no cooling channels.

The single cooling channel variant (square shaped and rounded) and version without them both had different values of mentioned parameters due to differences in geometry. It is worth mentioning that the type of settings remained the same. The comparison of values is presented in Table 2 and finalized meshes are shown in Figure 11. This figure shows areas of interest with increased mesh density. This approach allows for a higher accuracy of results.

#### 2.3.5. Simulation and Material Settings

In the presented study, only the part of the injection process was analyzed; to be more specific, only the wax cooling inside the cavity after injection. Therefore, the geometry elements, such as the injection channel, are removed to reduce the complexity of the simulation and decrease solving time. Such an element will not contribute to the simulation’s results in a significant way due to its low volume. Other omitted parts are bolts secured to stack up plates. Again, the reason for this action is to reduce the complexity of the computational model. In our physical model, the bolts and nuts are not in contact with plates. Those elements are isolated by plastic washers and air gaps. Hence, their impact on wax cooling is neglectable.

The important factor in the presented results is the utilization of isotropic material models for solid parts (including the wax model). This approach is based on experimental data gathered from material samples.

**Figure 12:**
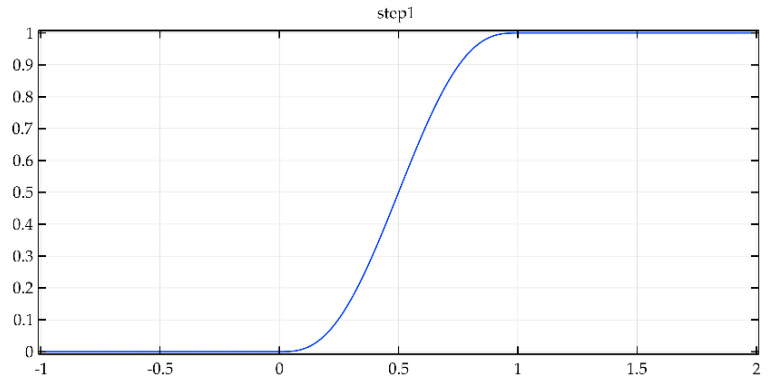
The step function graph used in simulation.

The presented study is not stationary problem, and thus must be investigated as transient. The characteristic feature of such an approach is the change in values of BC over time. The solver requires an input of time range with time step size. The total simulated time is 100 s. By default, the time steps taken by solver are calculated based on total simulation time. Most time-dependent problems solved by Comsol Multiphysics use the adaptative time-stepping theme. This means that timestep is adjusted to maintain requested relative tolerance. In this particular example, a well-known implicit solver, i.e., the backward differentiation formula (BDF) was used [54]. It uses backward differentiation formulas with varying order of accuracy. The biggest advantage of this method is its high stability and versatility. However, there is a downside to this method, in that it introduces higher damping at higher frequencies. The step function (Figure 12) was defined to increase the stability of simulation. Due to steady and continuous increase of flow rate and change of temperature, all BCs discontinuities are eliminated [55].

The whole description of simulation setting omitted material assignment to particular domains. All solid materials used in simulation are isotropic materials with the same properties in each direction. Their properties were obtained from experimental data and compared with literature. Some properties change depending on the temperature, and this was included in the material definition in Comsol Multiphysics by a linear piecewise function that approximated values in between measured ones. The materials used for fluids are imported from Comsol Material Library. Both water and air have properties that change with the domain of temperature.

## 3. Results and Discussion

**Figure 13:**
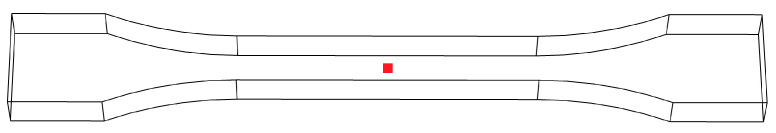
Location of the temperature measurement point (middle point).

The results of the simulation of the cooling process are presented below. The wax injection molds were assumed to be made in the SLA 3D printing technology from a photopolymer resin whose measured and averaged value of thermal conductivity was 0.53 W/m∙K, assuming a constant specific heat value of 1296 J/kg∙K. The wax injection mold was filled with wax with the following parameters: 3271 J/kg∙K (specific heat) and 0.37 W/m∙K (Table 1) in the case of thermal conductivity. The wax injection mold made of aluminum with thermal parameters of 900 J/kg∙K and 238 W/m∙K was adopted as a reference element. Designed geometry of the wax injection mold enables the production of samples used in the strength test, for which the middle point of the measuring section (Figure 5—*l*_0_) is the key factor. For this reason, the analysis of heat transfer at this middle point of the sample (Figure 13) and for the average temperature of the sample was performed.

### 3.1. The Measured Thermal Parameters of Materials

#### 3.1.1. Thermal Conductivity

In accordance with Section 2.2.1, the measurements were carried out in a steady state. Measurements were made at three selected temperatures of 40 °C, 50 °C, and 60 °C, in which the material does not change its properties. The results are presented in Table 3.

#### 3.1.2. The Specific Heat

The values of specific heat measurements were obtained with method described in Section 2.2.2. The determination of thermal conductivity was carried out in specified range of temperatures (from 40 °C to 60 °C). Thermometers with high measuring accuracy and precise laboratory balances were used. The tests were carried out according to the calorimeter operating procedures [56,57].

The measurements results are: 3271 J/kg∙K for wax and 1296 J/kg∙K for Rigid 10 K photopolymer resin.

### 3.2. Wax Injection Molds without Cooling Channels

**Figure 14:**
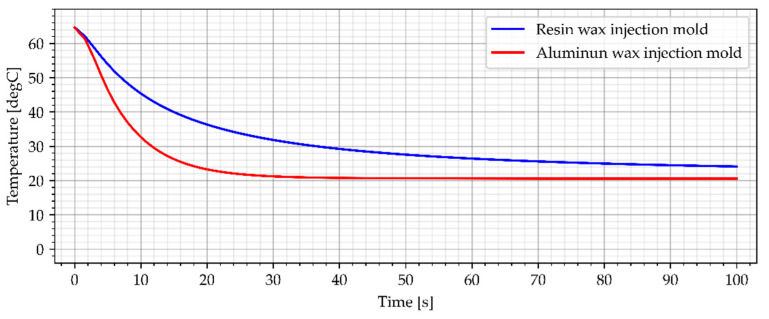
The sample temperature for wax injection molds without cooling channels (middle point).

**Figure 15:**
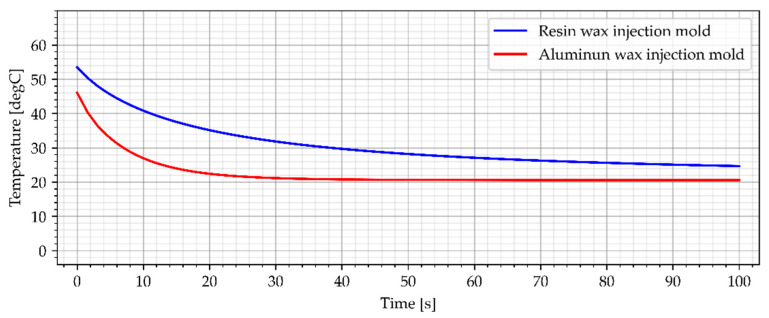
The average temperature of the sample for wax injection molds without cooling channels.

In the industrial approach of Investment Casting, wax injection molds are made of copper and aluminum alloys [12]. This is due to the necessity to make a large number of casting patterns, necessary to make the casting molds. As a result, one of the most important criteria for selecting a material for a wax injection mold is heat dissipation [12]. The cooling process of the reference aluminum wax injection mold in combination with the resin wax injection mold is presented in Figure 14 and Figure 15. Both wax injection molds did not have cooling channels. Such an approach allows for a direct comparison of the colling effectiveness of each material as the geometries are identical (only material was different).

### 3.3. Wax Injection Molds with Different Variation of Cooling Channel

#### 3.3.1. Water Cooling

**Figure 16:**
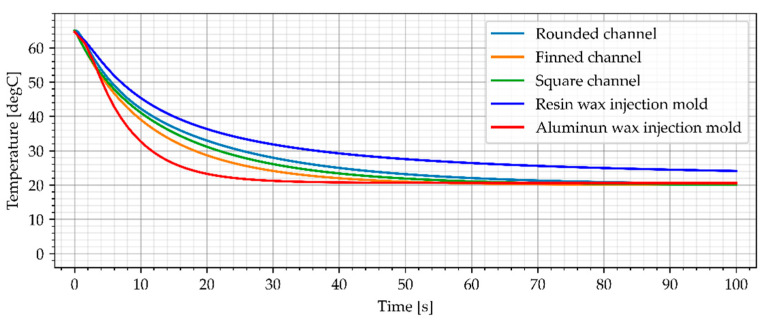
The sample temperature for water cooled wax injection molds (middle point).

**Figure 17:**
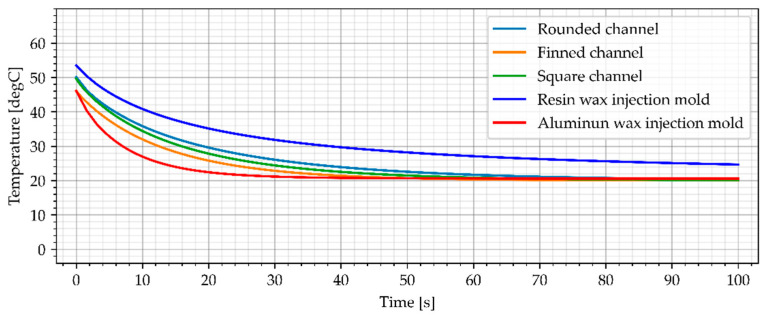
The average temperature of the sample.

One of the most commonly used cooling media in the traditional approach to the injection process is water. The simulation results for water (20 °C) cooled wax injection molds with the flow rate of 0.02 kg/s and the average temperature of the sample are shown in Figure 16 and Figure 17.

The temperatures of the sample (the average and center point values) for the water-cooled wax injection mold were contained between the reference wax injection molds. The cooling process of the resin wax injection mold lowered the temperature of the samples compared to the reference resin injection mold (without cooling channels). However, for the assumed range of the injection process determined by the minimum sample temperature of 40 °C (313K), the values are still higher than those of the aluminum wax injection mold. The temperature of the cooled resin wax injection molds equalizes with the traditional wax injection mold after about 80 s (40 s longer than the reference wax injection mold temperature reached).

#### 3.3.2. Air Cooling, Flow Rate of 0.0003 kg/s

**Figure 18:**
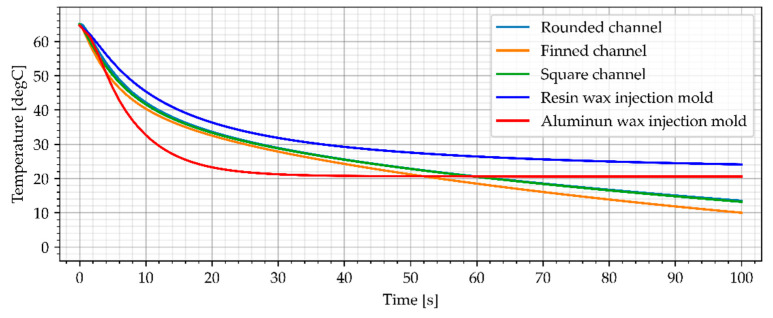
The sample temperature for air cooled wax injection molds (middle point).

**Figure 19:**
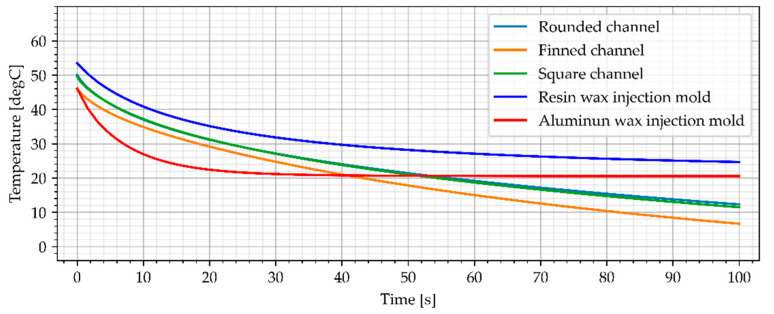
The average temperature of the sample.

The second cooling medium used in this study was cold air (−23 °C), which can be achieved using vortex cooling nozzles. The first variant included a slow flow rate of 0.0003 kg/s. The simulation results for air cooled wax injection molds and the average temperature of the sample are shown in Figure 18 and Figure 19.

The simulation results show that resin wax injection molds cooled with cold air can achieve lower temperatures that the reference aluminum wax injection mold after min. 40 s (average temperature of the sample). Moreover, as assumed, the wax injection mold with finned cooling channels was the best cooling system. After 60 s, all the air-cooled resin wax injection molds had a lower temperature of the samples (middle point and average temperature) than the aluminum wax injection mold.

#### 3.3.3. Air Cooling, Flow Rate of 0.0006 kg/s

**Figure 20:**
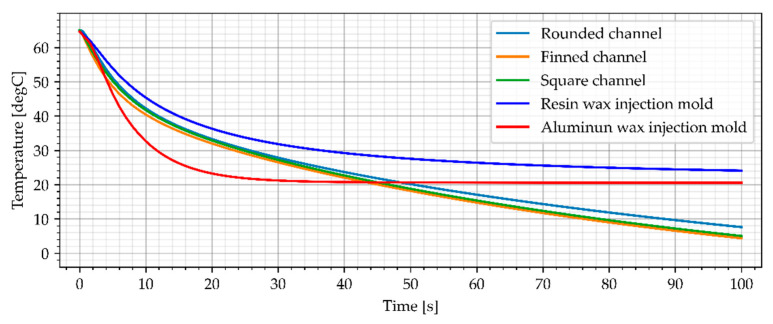
The sample temperature for air cooled wax injection molds (middle point).

**Figure 21:**
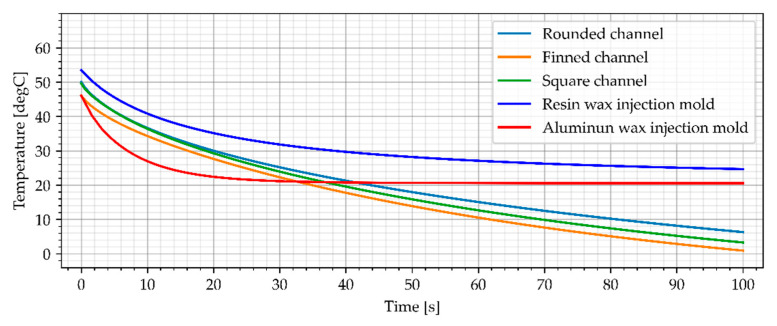
The average temperature of the sample.

The third simulation variant takes into account the cold air (−23 °C) flow rate of 0.0006 kg/s. The simulation results for air cooled wax injection molds and the average temperature of the sample are shown in Figure 20 and Figure 21.

The simulation results show that doubling the cold air flow resulted in a lower samples temperature. The average temperature of all samples was lower than those obtained in the reference aluminum wax injection mold after 42 s (34 s for the finned version). The time difference needed to achieve better results for the cooled wax injection molds compared to the reference aluminum wax injection mold was not significant and amounted to between 44s for the finned channel and 48 s for rounded version.

#### 3.3.4. Air Cooling, Flow Rate of 0.001 kg/s

**Figure 22:**
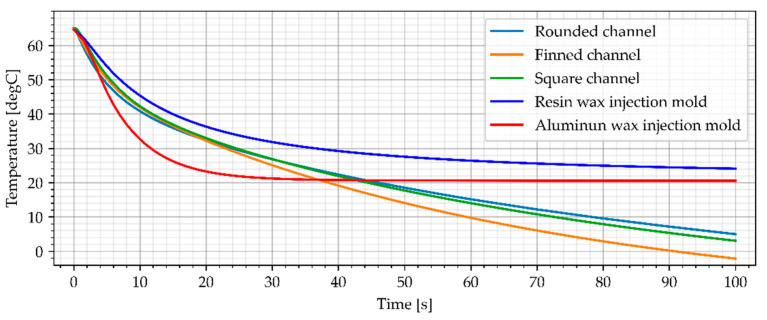
The sample temperature for air cooled wax injection molds (middle point).

**Figure 23:**
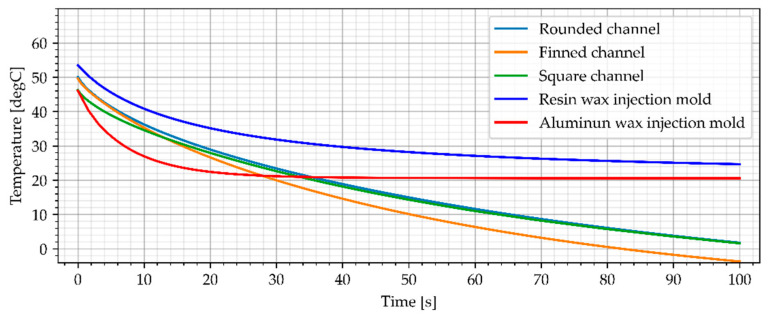
The average temperature of the sample.

The last simulation variant takes into account the cold air (−23 °C) flow rate of 0.001 kg/s. The simulation results for air cooled wax injection molds and the average temperature of the sample are shown in Figure 22 and Figure 23.

Cooling the resin wax injection molds with cold air (−23 °C) with a flow of 0.001 kg/s resulted in achieving the best results among all simulations. The finned cooling channel is characterized by the best parameters - reduced the time needed to cool the sample below the reference value to 28 s (average temp) and 37 s (the middle point). The other types of cooling channels also noticeably shortened the time needed to cool the samples.

### 3.4. Temperature Distribution in Wax Injection Mold

**Figure 24:**
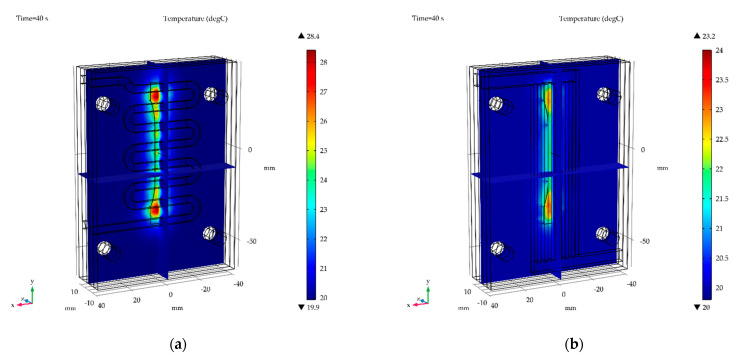
The temperature distribution in wax injection mold for the water cooling variant with: (**a**) rounded channels, (**b**) finned channels.

**Figure 25:**
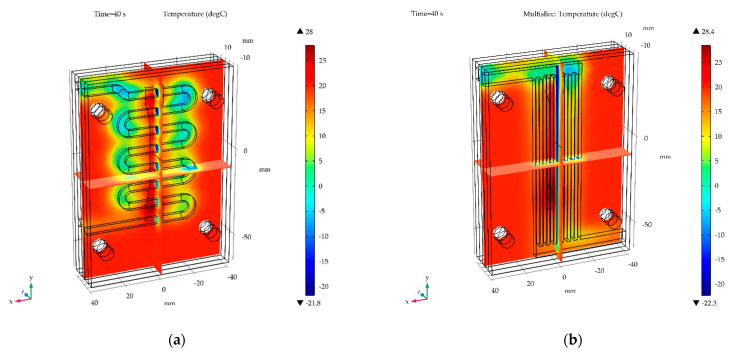
The temperature distribution in wax injection mold for air cooled with flow rate of 0.001 kg/s with: (**a**) rounded channels, (**b**) finned channels.

The temperature distribution in wax injection mold after 40s for water cooled (the worst variant) and air cooled with flow rate of 0.001 kg/s (the best variant) is shown in Figure 24 and Figure 25, respectively.

### 3.5. Results Summary

This study presents an analysis of the possibilities of improving the thermal conditions of the manufacturing process of wax models in a 3D printed wax injection mold. The main task was to prove that despite the very low thermal parameters of the mold material, unfavorable to the process, it is possible to shorten the time needed to produce a single wax model. This study focused on the analysis of methods of cooling the 3D printed wax injection mold made of photopolymer resin in the SLA technology. SLA technology, due to its accuracy, provides comparable geometric and dimensional parameters to metal alloys made in subtractive technologies.

The first stage of the research included the determination of key thermal parameters of individual materials used in this process. Due to trade secrets regarding the exact chemical composition of individual materials, manufacturers do not provide these parameters, and without them it is impossible to obtain reliable simulation results.

In the second stage of these tests, a cooling simulations of a reference wax injection molds without any cooling channels made of pure aluminum alloy and photopolymer resin ware simulated. It is worth emphasizing that in the foundry industry, due to the ease of processing and availability, aluminum-silicon alloys are often used (not pure aluminum), which are characterized by lower thermal parameters (in special cases, even 3 times lower).

The last phase of the research involved simulation for three types of cooling channels (rounded, square and finned) and two cooling media (water and cold air). In order to analyze the possibility of cooling the wax injection molds, the average temperature of the sample and due to the geometry of the analyzed sample (strength sample), the temperature in the middle point (the most important point in the strength test) of the sample was selected.

Analyzing the simulation results allowed to select the best variant of cooling of the 3D printed resin wax injection mold. Detailed middle point temperatures for each variant over time are presented in Table S1, while the detailed sample average temperatures for the different variants over time are shown in Table S2.

Analysis of the results for the middle point temperature of the sample presented in the graphs and Table S1 showed that in all cases, after reaching the specified time, the best results are achieved by air cooling with the flow of 0.001 kg/s. For all types of cooling channels, in the case of the middle point temperature, the water absorbed heat best in the first stage of cooling. For finned and square cooling channel it took 30 s, while for the rounded it was only 25 s. The reference resin wax injection mold had the worst performances in all cases. The best cooling results compared to the aluminum reference wax injection mold for middle point analysis were achieved by using finned cooling channels with air cooling (flow of 0.001 kg/s) after 40s (20.8 °C vs. 19.1 °C). These results show that the mean break point for middle point temperature is reached after about 35–40 s for cold air cooling with the highest analyzed flow.

Analysis of the results for the average temperature of the sample presented in the graphs and Table S2 showed that in all cases, after reaching the specified time, the best results are achieved by air cooling with the flow of 0.001 kg/s as well as in the middle point analysis. For all types of cooling channels, in the case of the average temperature of the sample, the water absorbed heat best in the first stage of cooling. For finned and square cooling channel it took 25 s, while for the rounded it took as long as 35s (the worst variant). The average temperature of the sample in the reference wax injection mold again had the highest levels. The best cooling results compared to the aluminum reference wax injection mold for average temperature of the sample were achieved by using finned cooling channels with air cooling (flow of 0.001 kg/s) after 30s (21.2 °C vs. 20.0 °C). These results show that the mean break point for average temperature of the sample is reached after about 30s for cold air cooling with the highest analyzed flow.

## 4. Conclusions

The main objective of this study was the heat transfer analysis of the 3D printed photopolymer wax injection mold with the use of various cooling media and different geometries of cooling channels. Based on the results discussed in this study, the following conclusions can be drawn:

1. Water at room temperature has the best ability to receive heat in the first stage of cooling process (about 25 s).
2. After reaching the temperature from point 1, cold air cooling with highest flow rate of 0.001 kg/s is the most effective process.
3. After about 30–35 s, air cooling becomes more effective than heat dissipation through aluminum material.
4. The finite element method (FEM) analysis used in this study can be successfully used to carry out thermal analyses, thanks to which it is much easier to plan the production process (e.g., calculation of production capacity). However, it should be emphasized that errors in result estimation can occur. Hence, experimental validation of obtained results in crucial.
5. The 3D printed wax injection molds, under certain conditions, can be successfully used in the production process. However, in order to accelerate production, it is worth using cooling channels, which can significantly speed up the production process. This approach appears to present an ideal substitute for traditional wax injection molds for low-volume production which, moreover, does not require additional, new materials in the production flow.

The directions of future research should include verification of the obtained results in the real environment, maintaining the current boundary conditions.

## Figures and Tables

**Table 1:** Dimensions of the sample ISO EN ISO 527-2:2012 [49].

Name	No.	Value [mm]
Total length	*l*_3_	≥75
The length of the part delimited by parallel lines	*l*_1_	30.0 ± 0.5
Radius	*r*	≥30
The distance between wide parallel parts	*l*_2_	58 ± 2
Width at the ends	*b*_2_	10 ± 0.5
Width of the narrow part	*b*_1_	5.0 ± 0.5
Thickness	*h*	≥2
Length of the measuring section	*l*_0_	10 ± 0.2
Initial distance between the handles	*l*	l_2_ (−0, +2)

**Table 2:** The comparison of mesh values.

Setting Name	Finned Version	Square Channel	Rounded Channel	No Channels
max. element size (laminar flow) [mm]	3.00	2.94	2.94	NA
min. element size (laminar flow)[mm]	0.60	0.88	0.88	NA
max. growth rate (laminar flow) [mm]	1.15	1.15	1.15	NA
max. element size (solid)[mm]	4.00	6.16	6.16	3.92
min. element size (solid) [mm]	0.40	0.45	0.45	0.17
max. growth rate (solid) [mm]	1.35	1.40	1.40	1.35
max. element size (boundaries) [mm]	1.00	2.94	2.94	NA
min. element size (boundaries) [mm]	0.08	0.88	0.88	NA
max. growth rate (boundaries) [mm]	1.10	1.15	1.15	NA
min. skewness of elements	0.41	0.28	0.22	0.34
min. volume versus length	0.43	0.38	0.37	0.38
Number of elements	113,916	134,418	184,703	64,812

**Table 3:** The measured values of thermal conductivity.

Property	Temperature	Value	Unit
Thermal conductivity of wax	40 °C	0.35	W/(m K)
	50 °C	0.37	
	60 °C	0.39	
Thermal conductivity of resin	40 °C	0.50	W/(m K)
	50 °C	0.53	
	60 °C	0.55	

## Data Availability

The data available on request.

## Supplementary Materials

The following supporting information can be downloaded at: https://www.mdpi.com/article/10.3390/ma15196545/s1, Table S1: Sample middle point temperature; Table S2: Average sample temperature.
