# Supplementary material for: Heat Transfer Analysis of 3D Printed Wax Injection Mold Used in Investment Casting
Source: Materials (Basel). 2022 Sep 21;15(19):6545. doi: 10.3390/ma15196545 (PMC9571762; doi:10.3390/ma15196545)
Supplement: Supplementary file 1 [file materials-15-06545-s001.zip › materials-1898973-supplementary.pdf]

Table S1

Model variant	Cooling medium	Sample middle point temperature [°C]															
		Time [s]															
		5	10	15	20	25	30	35	40	45	50	55	60	65	70	75	80
Aluminium without channels	n.a.	46,4	32,7	26,2	23,3	21,9	21,2	20,9	20,8	20,7	20,7	20,6	20,6	20,6	20,6	20,6	20,6
Resin without channels	n.a.	53,9	45,4	40,0	36,3	33,7	31,8	30,4	29,2	28,3	27,6	26,9	26,4	26,0	25,6	25,3	25,0
Finned	water [20°C] - 0.02 kg/s	49,1	39,1	32,8	28,7	25,9	24,1	22,8	22,0	21,4	21,0	20,7	20,5	20,3	20,2	20,2	20,1
	air [-23°C] - 0.0003 kg/s	48,5	40,3	35,7	32,5	30,0	27,8	26,0	24,2	22,7	21,2	19,8	18,5	17,3	16,1	14,9	13,9
	air [-23°C] - 0.0006 kg/s	48,6	40,4	35,6	32,0	29,1	26,5	24,2	22,0	20,0	18,2	16,4	14,8	13,2	11,7	10,3	9,0
	air [-23°C] - 0.001 kg/s	48,8	39,9	35,5	32,2	28,5	25,1	22,0	19,1	16,5	14,1	11,8	9,8	7,8	6,1	4,4	2,9
Square channel	water [20°C] - 0.02 kg/s	50,0	41,1	35,2	31,2	28,2	26,1	24,5	23,4	22,5	21,9	21,4	21,1	20,8	20,6	20,5	20,3
	air [-23°C] - 0.0003 kg/s	50,2	41,6	36,7	33,3	30,8	28,8	27,0	25,5	24,1	22,8	21,6	20,5	19,4	18,4	17,5	16,5
	air [-23°C] - 0.0006 kg/s	50,2	41,7	36,6	32,9	29,8	27,2	24,9	22,7	20,7	18,8	17,0	15,4	13,8	12,4	11,0	9,6
	air [-23°C] - 0.001 kg/s	50,3	40,8	35,9	32,4	29,4	26,8	24,5	22,4	20,4	18,5	16,8	15,2	13,7	12,2	10,9	9,6
Rounded channel	water [20°C] - 0.02 kg/s	51,2	42,3	36,8	33,0	30,1	28,0	26,3	25,0	24,0	23,2	22,5	22,0	21,6	21,3	21,1	20,9
	air [-23°C] - 0.0003 kg/s	51,2	42,2	37,1	33,6	31,0	28,9	27,1	25,5	24,1	22,9	21,7	20,6	19,5	18,5	17,6	16,7
	air [-23°C] - 0.0006 kg/s	51,1	42,3	37,0	33,3	30,4	27,9	25,7	23,7	21,9	20,2	18,6	17,1	15,7	14,4	13,1	11,9
	air [-23°C] - 0.001 kg/s	51,2	42,3	36,9	33,0	29,7	26,9	24,3	21,9	19,7	17,7	15,8	14,0	12,4	10,8	9,3	7,9

Table S2

Model variant	Cooling medium	Average sample temperature [°C]															
		Time [s]															
		5	10	15	20	25	30	35	40	45	50	55	60	65	70	75	80
Aluminium without channels	n.a.	32,8	27,0	24,0	22,4	21,6	21,2	20,9	20,8	20,7	20,7	20,6	20,6	20,6	20,6	20,6	20,6
Resin without channels	n.a.	45,6	40,8	37,6	35,2	33,3	31,8	30,7	29,7	28,9	28,2	27,6	27,1	26,7	26,3	25,9	25,6
Finned	water [20°C] - 0.02 kg/s	37,4	32,0	28,3	25,8	24,1	22,8	22,0	21,4	21,0	20,7	20,5	20,4	20,2	20,2	20,1	20,1
	air [-23°C] - 0.0003 kg/s	38,9	34,9	31,8	29,2	26,8	24,7	22,8	21,0	19,4	17,8	16,4	15,0	13,8	12,6	11,4	10,4
	air [-23°C] - 0.0006 kg/s	38,7	34,3	30,7	27,6	24,8	22,3	19,9	17,8	15,8	13,9	12,2	10,5	9,0	7,6	6,3	5,1
	air [-23°C] - 0.001 kg/s	38,9	34,2	30,6	26,6	23,1	20,0	17,2	14,6	12,3	10,1	8,2	6,4	4,7	3,2	1,8	0,5
Square channel	water [20°C] - 0.02 kg/s	40,1	34,4	30,6	27,8	25,9	24,4	23,3	22,5	21,9	21,5	21,1	20,9	20,7	20,5	20,4	20,3
	air [-23°C] - 0.0003 kg/s	41,6	37,2	33,9	31,2	29,0	27,1	25,4	23,8	22,4	21,1	19,8	18,7	17,6	16,6	15,7	14,7
	air [-23°C] - 0.0006 kg/s	41,4	36,4	32,5	29,3	26,4	23,9	21,7	19,6	17,6	15,9	14,2	12,7	11,2	9,9	8,6	7,4
	air [-23°C] - 0.001 kg/s	41,0	34,6	31,0	27,9	25,2	22,6	20,3	18,1	16,1	14,3	12,6	11,0	9,6	8,2	7,0	5,8
Rounded channel	water [20°C] - 0.02 kg/s	29,6	27,6	26,1	24,9	23,9	23,2	22,6	22,1	21,7	21,4	21,1	20,9	20,8	20,6	20,5	20,4
	air [-23°C] - 0.0003 kg/s	41,6	37,0	33,8	31,2	29,0	27,1	25,5	24,0	22,7	21,4	20,2	19,2	18,1	17,2	16,3	15,4
	air [-23°C] - 0.0006 kg/s	41,5	36,6	33,0	30,0	27,5	25,2	23,2	21,3	19,6	18,0	16,5	15,1	13,8	12,5	11,3	10,2
	air [-23°C] - 0.001 kg/s	41,4	36,2	32,3	28,9	26,0	23,4	21,0	18,9	16,9	15,0	13,3	11,6	10,1	8,7	7,3	6,1
